# Prediction of Emerging CVD Biomarkers in Population Exposed to Arsenic and Their Associated Risk Factors in Humans by Systematic Review and Meta-Analysis

**DOI:** 10.1007/s12011-025-04674-2

**Published:** 2025-06-10

**Authors:** Noorul Izzati Hanafi, Mohd Hafiz Dzarfan Othman, Siti Hamimah Sheikh Abdul Kadir

**Affiliations:** 1 Cardiovascular Advancement and Research Excellence Institute (CARE Institute), Universiti Teknologi MARA, Campus Sungai Buloh, Jalan Hospital, Sungai Buloh, 47000 Selangor, Malaysia; 2 Advanced Membrane Technology Research Centre (AMTEC), Universiti Teknologi Malaysia, 81310 Skudai, Johor, Malaysia; 3 Biochemistry and Molecular Medicine Department, Faculty of Medicine, Universiti Teknologi MARA, Sungai Buloh, Malaysia 47000, Jalan Hospital, Selangor

**Keywords:** Arsenic, Cardiovascular disease, Risk factors, Biomarkers

## Abstract

Arsenic is a natural compound of metalloid (both metal and non-metal) found in soil and minerals used in semiconductor and alloys. Arsenic exposure is associated with an increased risk of cardiovascular disease (CVD) risk from drinking water or environmental sources. Exploring the emerging biomarkers associated with CVD upon arsenic exposure is crucial. Thus, this review paper aimed to analyze the prediction of emerging CVD biomarkers in population exposed to arsenic and their associated risk factors in humans by a systematic review and meta-analysis. A systematic search was conducted using PubMed, Scopus, and Web of Science to identify eligible studies on arsenic exposure associated with CVDs and their risk factors. Sensitivity, specificity, and predictive value of biomarkers for CVD of arsenic exposed population were assessed by calculating the quality criteria, standard means different (SMD), odds ratio (OR), and 95% confidence intervals (CIs). A meta-analysis was used to derive a combined SMD and OR for the heterogeneity test in between studies. The meta-analysis of nine articles were included. The overall effect sizes revealed significant heterogeneity across studies (*I*^2^ = 97.57%), with pooled effect size was 3.578 (95% CI 3.032 to 4.123, *p* < 0.0001) under the random-effects model. These results indicate a robust association between arsenic exposure and adverse cardiovascular outcomes, as confirmed by sensitivity analyses and bias assessments (Systematic review registration: PROSPERO CRD42024616848). To improve the quality of future research, efforts should concentrate on enhancing control for confounders, enrolling diverse participant populations, and implementing and yield more substantial findings concerning the cardiovascular effects of arsenic exposure.

## Introduction

Cardiovascular disease (CVD) continues to pose a significant global public health burden, serving as the leading cause of mortality and morbidity worldwide [29]. Despite progress in medical interventions and preventive measures, the prevalence of CVD is rising, largely due to modifiable risk factors such as hypertension, dyslipidemia, diabetes, and environmental toxin exposure [1]. Among these, arsenic, a naturally occurring metalloid, has emerged as a key environmental factor associate with an increase of CVD risk, particularly in populations exposed through contaminated water or industrial emissions [15]. The complex toxicological effects of arsenic on cardiovascular health necessitate further investigation into its interactions with both established and emerging CVD biomarkers.

Emerging biomarkers of cardiovascular health, encompassing those associated with inflammation, oxidative stress, endothelial dysfunction, and metabolism, are increasingly recognized for their role in refining CVD risk assessment and management [26]. These biomarkers enhance understanding of disease mechanisms and support early interventions [27]. Their identification is critical in populations exposed to arsenic, as cardiovascular effects are mediated by oxidative stress, inflammation, and epigenetic modifications, which collectively drive the development and progression of CVD [8].

Chronic arsenic exposure, primarily through contaminated drinking water and industrial processes, remains a pressing issue in low- and middle-income countries, where water quality regulations are often insufficient [30]. Long-term exposure of arsenic has been associated with elevated risks of CVD, including coronary artery disease, peripheral arterial disease, and cerebrovascular events [16]. Mechanistically, arsenic induces oxidative stress, disrupts endothelial function, and provokes systemic inflammation, all of which are critical drivers of atherosclerosis and thrombosis [4, 24]. Additionally, arsenic interferes with lipid and glucose metabolism and contributes to metabolic disorders that lead to CVD risk [22]. Epidemiological research demonstrates a dose-dependent relationship between arsenic exposure and adverse cardiovascular outcomes. Studies from highly affected regions, such as Bangladesh, reveal strong associations between elevated arsenic levels in drinking water and increased CVD prevalence [2]. However, individual susceptibility varies, and as a result, the predictive capability of biomarkers for cardiovascular outcomes in arsenic-exposed populations also differs [13]. The interplay between arsenic toxicity and traditional CVD risk factors further complicates risk stratification, highlighting the importance of integrating emerging biomarkers into clinical frameworks.

Emerging biomarkers offer valuable insights into arsenic-induced cardiovascular risk, addressing key biological processes such as inflammatory markers. Biomarkers such as high-sensitivity C-reactive protein, interleukin-6, and tumor necrosis factor-alpha are established predictors of cardiovascular events [25]. Oxidative stress biomarkers elevated in arsenic exposure by promoting reactive oxygen species production, lead to lipid peroxidation and protein damage [32]. Biomarkers such as malondialdehyde, 8-isoprostane, and oxidized low-density lipoprotein reflect oxidative damage linked to CVD [3] Furthermore, endothelial dysfunction markers that are arsenic-related oxidative stress and nitric oxide (NO) imbalance contribute to an endothelial dysfunction, an early step in atherosclerosis. Indicators such as asymmetric dimethylarginine (ADMA), soluble intercellular adhesion molecule-1, and vascular endothelial growth factor (VEGF) are associated with endothelial health [19].

Moreover, arsenic is reported to disrupt diabetes and metabolic syndrome biomarkers such as lipid metabolites [23]. Biomarkers like advanced glycation end products, adiponectin, and apolipoproteins capture these metabolic disturbances and their links to CVD [20]. Epigenetic markers such as advancement in epigenomics have identified arsenic-induced modifications, including changes in DNA methylation, histone modifications, and microRNA expression [21]. Biomarkers such as DNA methylation patterns in CVD-related genes and circulating microRNAs (as example miR-21, miR-126) offer insights into arsenic-mediated cardiovascular toxicity [28].

Incorporating emerging biomarkers into CVD risk assessment frameworks can significantly improve the understanding and management of arsenic-induced cardiovascular effects [14]. These biomarkers facilitate early detection of subclinical damage, support targeted interventions, and aid in monitoring therapeutic responses. Moreover, they can guide public health initiatives to reduce arsenic exposure. Notwithstanding, their potential, certain obstacles impede the practical application of these biomarkers, such as the necessity for assay standardization, validation across heterogenous populations, and the economic burden of testing. The impact of genetic, nutritional, and socioeconomic factors on arsenic poisoning is significant.

Arsenic exposure has been linked to an increased risk of CVD; yet, the scope and mechanisms of this association remain unclear. This study systematically evaluates the variability in findings from previous research and identifies key confounding factors that may influence the relationship between arsenic exposure and cardiovascular risk. By assessing heterogeneity using statistical methodologies, it explores the role of potential confounders in arsenic-related CVD outcomes and examines how differences in study designs impact results. A more comprehensive understanding of these factors is essential for advancing research methodologies and shaping targeted prevention efforts, particularly in high-exposure regions.

## Methodology

This systematic review and meta-analysis aimed to identify, evaluate, and synthesize findings from cohort studies, case–control studies, randomized controlled trials (RCTs), and observational studies that investigated the role of emerging cardiovascular biomarkers in individuals exposed to arsenic. The focus was on populations with documented CVD risk and confirmed arsenic exposure, assessing how emerging biomarkers compare with traditional markers in predicting cardiovascular events, early disease progression, and biomarker trends.

### Eligibility Criteria

Studies were eligible for inclusion if they involved human participants with confirmed arsenic exposure from environmental, occupational, or lifestyle sources and assessed emerging cardiovascular biomarkers in relation to CVD risk. These biomarkers included inflammatory markers, cardiac-specific indicators, oxidative stress markers, and measures of endothelial function. Eligible studies are also needed to report either quantitative or qualitative cardiovascular outcomes or compare biomarker levels between arsenic-exposed and unexposed groups. Only studies employing observational designs (cross-sectional, cohort, or case–control) or randomized controlled trials were considered. Studies were excluded if the involved participants without documented arsenic exposure or cardiovascular risk, focused solely on general CVD risk factors without assessing biomarkers, lacked relevant cardiovascular outcomes, or did not include primary data. Additionally, case reports, editorials, review articles, and studies conducted on animals, plants, or in vitro models were excluded from this review.

### Search Strategy

The literature search was conducted using PubMed, Scopus, and Web of Science, targeting English-language studies. The search was finalized in December 2024. To ensure a thorough coverage, the protocol was registered with PROSPERO (CRD42024616848) in accordance with PRISMA-P guidelines [12]. Manual reference checks and expert consultations were also conducted to identify any additional relevant studies.

### Study Selection and Data Extraction

**Fig. 1:**
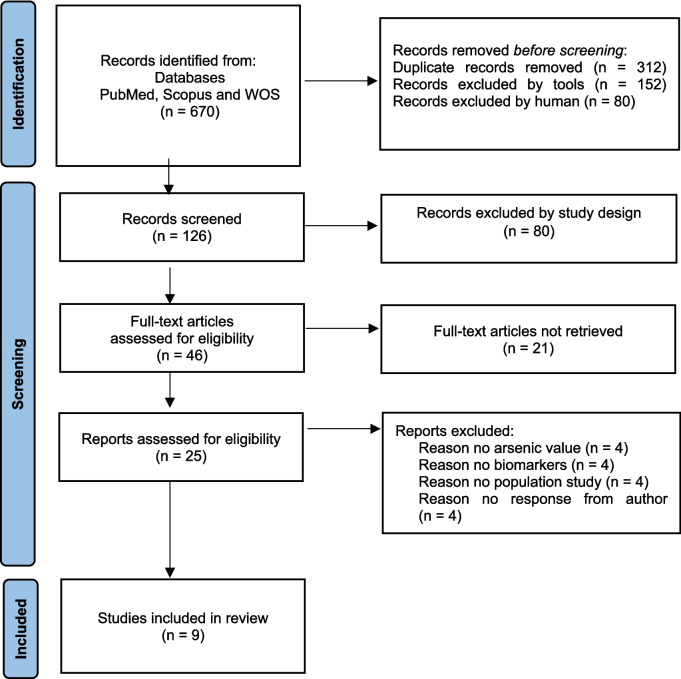
The flow of study identification and selection

Two independent reviewers (NIH, SHSAK) screened titles, abstracts, and full texts. Disagreements were resolved by a third reviewer (MHDO). The selection process followed PRISMA guidelines and was documented using a flow diagram (Fig. 1). Data were extracted using a piloted form capturing study characteristics, intervention details, participant demographics, exposure assessment, biomarker data, and reported cardiovascular outcomes.

### Quality Assessment and Risk of Bias

Study quality was assessed using two tools, the Cochrane Risk of Bias tool for randomized trials and the Newcastle–Ottawa Scale for observational studies. These evaluations were conducted independently by NIH and SHSAK. The Cochrane tool assessed selection, performance, detection, attrition, and reporting bias, while the Newcastle–Ottawa Scale evaluated selection, comparability, and outcome or exposure assessment. Based on these assessments, studies were classified as low, moderate, or high risk of bias.

### Language and Publication Bias

Only English-language studies were included, which may introduce language bias and limit global representativeness. However, several steps were taken to address this limitation. The databases used provided broad international coverage, and nine manuscripts from countries including Spain, China, Japan, and Italy were initially identified. These non-English studies were excluded not due to language but because they did not meet the inclusion criteria by which focusing on unrelated outcomes, non-human studies, or lacking arsenic exposure data.

### Adjustment for Confounding

Confounding was addressed through a subgroup analysis and adjustment strategies within the included studies. As example, adjustments were made for key confounders such as age, which was notably important in the observed associations between the left ventricular (LV) mass and carotid intima-media thickness (cIMT). Subgroup analyses served as exploratory functions, helping to identify variables that could influence the relationship between arsenic exposure and cardiovascular biomarkers. This approach ensured that associations reflected true relationships rather than external influences, ultimately strengthening the validity of the findings.

This meta-analysis explored the link between environmental arsenic exposure and CVD risk, using data from studies that measured arsenic from sources like drinking water, groundwater, coal burning, or industrial pollution. Because exposure methods varied, the analysis focused on an overall environmental arsenic rather than just an inorganic arsenic (iAs). While a few studies did full arsenic breakdowns, most measured total urinary arsenic (∑As) or arsenic in environmental samples, in areas with low seafood consumption, and ∑As is a reasonable proxy for iAs and its metabolites (MMA, DMA).

### Missing Data

Missing data were managed by reaching out to study authors or sponsors for clarification or additional information. When unavailable, missing data were handled with caution and noted in the final interpretation of results.

### Statistical Analysis

Effect sizes were reported as standardized mean differences (SMD) for continuous outcomes. A random-effects model was planned and applied where appropriate. Heterogeneity was assessed using the *I*^2^ statistic, with values above 50% indicating substantial heterogeneity. *Z*-statistics were used to determine statistical significance, with *Z*-values greater than 1.96 considered significant at the 0.05 level.

## Results

The study selection process followed the PRISMA guidelines, as shown in Fig. 1. The initial search identified 670 records. After removing 544 records, including 312 duplicates and 232 considered ineligible by human and automated tools, 126 records were left for screening. During the screening phase, 80 are excluded by the study design. This left 46 full-text articles for further assessment. Thus, 21 articles were unavailable, leaving 25 for detailed evaluation. Twelve articles were excluded for reasons such as inconsistent arsenic values (*n* = 4), lack of relevance to biomarker outcomes (*n* = 4), and differences in study populations (*n* = 4). Finally, nine studies met the inclusion criteria and were included in the review [6, 7, 9–11, 18, 17, 19, 31].

The exclusion of certain studies was based on specific criteria to ensure the reliability and robustness of the meta-analysis. Studies with significant inconsistencies in arsenic exposure values were excluded, as variations in exposure assessment methods, such as discrepancies between urinary arsenic and drinking water arsenic measurements, could lead to biased results and distort the overall effect estimate. Additionally, studies were excluded if they did not focus on relevant cardiovascular biomarkers or if the populations studied differed substantially from the majority of included studies. Such differences in biomarkers or study populations could introduce heterogeneity or confounding, potentially compromising the interpretation of the relationship between arsenic exposure and cardiovascular disease risk. These exclusions were necessary to ensure a more consistent, focused analysis and enhance the validity of the conclusions drawn from the meta-analysis.

### Biomarkers Associated with CVD in Arsenic Exposure Populations

The current analysis shows an impact of arsenic exposure on CVD, emphasizing its influence on endothelial function, oxidative stress, lipid metabolism, cardiac structure, arsenic metabolism, and inflammatory responses. Endothelial dysfunction is reflected by elevated levels of ADMA and ICAM1, endothelial nitric acid synthase (eNOS), VEGF towards vascular damage, and disrupted nitric oxide signaling. Simultaneously, oxidative stress markers such as matrix metalloproteinases (MMP-2, MMP-9), telomere shortening, and mitochondrial DNA deletions are associated with cellular injury from arsenic exposure.

Dysregulation in lipid metabolism, characterized by elevated total cholesterol, triglycerides, low-density cholesterol levels, and altered high-density cholesterol ratios, with increased atherogenic potential is evidenced by a higher atherogenic index of plasma (AIP). Structural and functional cardiac changes, including increased left ventricular mass and wall thickness, suggest arsenic-induced hypertrophic remodeling. Variability in arsenic metabolism, indicated by urinary species (inorganic arsenic, methylmalonic acid, dimethylamine) and methylation profiles, highlights interindividual differences in susceptibility to arsenic toxicity and associated to CVD. Inflammatory markers such as miR-155 and fatty acid–binding protein (FABP4) further elucidate the systemic inflammation, underlying arsenic-related cardiovascular dysfunction.

In conclusion, the evidence highlights a complex effect of arsenic exposure on cardiovascular health, including endothelial dysfunction, oxidative stress, altered lipid metabolism, cardiac remodeling, and systemic inflammation. These interconnected mechanisms contribute to the increased cardiovascular disease risk linked to arsenic toxicity. Individual differences in arsenic metabolism emphasize the need for personalized risk assessment and intervention strategies. Implementing targeted measures could help in reduction of the arsenic exposure impact on the cardiovascular health and improve well beings in affected populations.

### Statistical Comparisons

A total of nine studies were included in the meta-analysis, investigating the relationship between arsenic exposure associated with CVD. The studies encompassed diverse biological mechanisms, including endothelial dysfunction, oxidative stress, inflammation, lipid metabolism, genetic regulation, and cardiac geometry.

### Analysis of Overall Effect Size and Heterogeneity

**Fig. 2:**
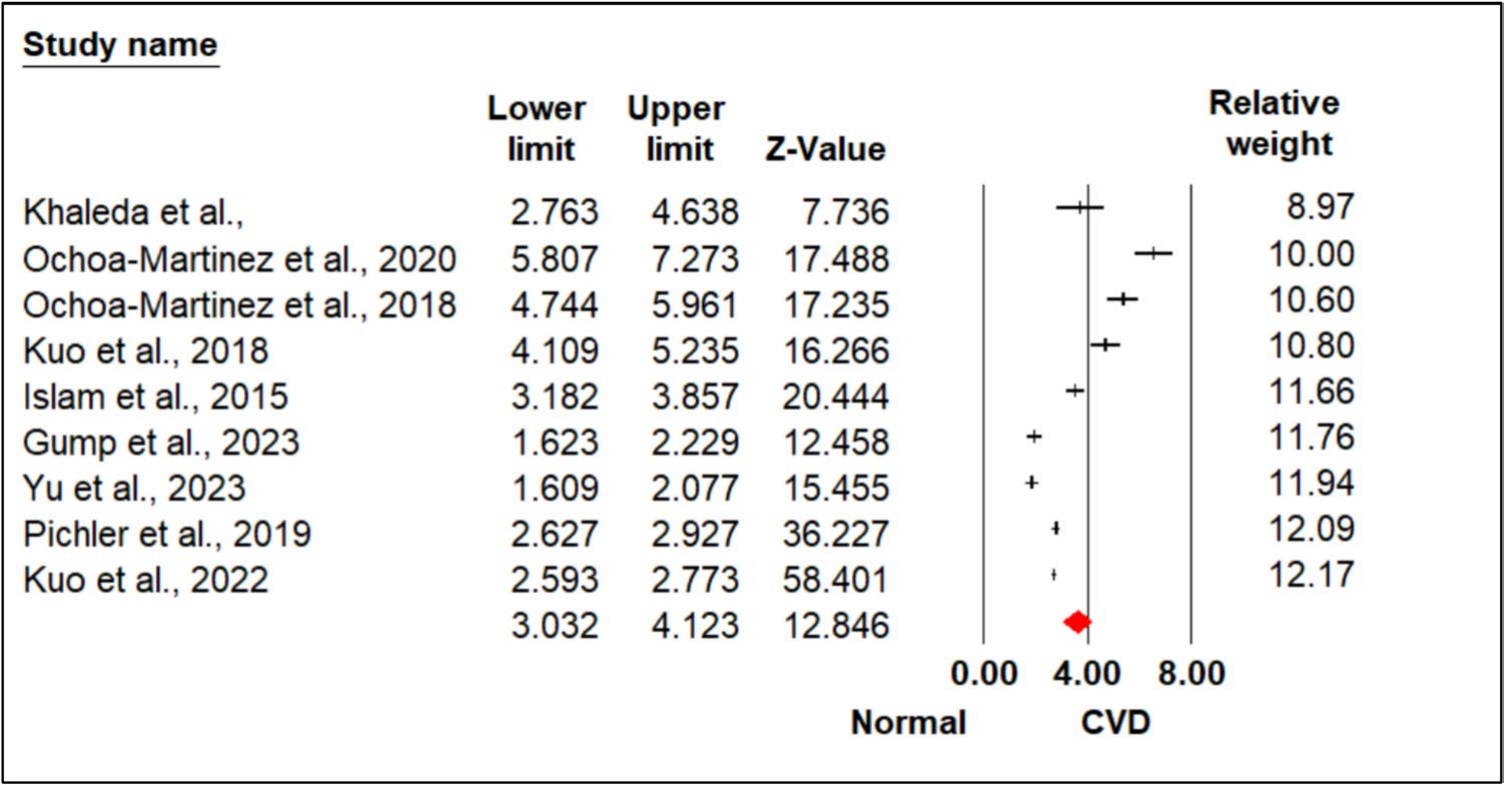
Heterogeneity represents by forest plot explained the association of overall effect size of arsenic-induced CVD. Meta-analysis using random-effects model, ordered by their relative weight. Test for heterogeneity: *I*.^*2*^ = 97.574%; *Q* = 329.805, overall effect, *Z* = 12.846

Figure 2 show the forest plot provided a detailed summary of the individual study contributions and the pooled effect sizes. The random-effects model yielded an SMD of 3.578 (95% CI 3.032 to 4.123, *p* < 0.0001) and chosen as a substantial heterogeneity that was evident (*Q* = 329.805, *p* < 0.001, *I*^2^ = 97.574%), justifying the use of the random-effects model to account for between-study variability.

### Analysis of Publication Bias on Overall Effect Size

The assessment of publication bias employed multiple statistical approaches and visualization techniques to ensure the reliability of the findings. Duval and Tweedie’s trim-and-fill method identified four potentially missing studies. Adjusting for these studies shifted the pooled effect size fixed-effects model, and the observed point estimate was 2.72842 (95% CI 2.66013 to 2.79670), which was adjusted to 2.62425 (95% CI 2.55733 to 2.69117) after accounting for four missing studies. Under the random-effects model, the observed point estimate was 3.57758 (95% CI 3.03171 to 4.12346), and after the adjustment, it was 3.59322 (95% CI 2.01899 to 3.16745). These adjustments suggest that missing studies have a minor impact on the overall effect size estimates.

**Fig. 3:**
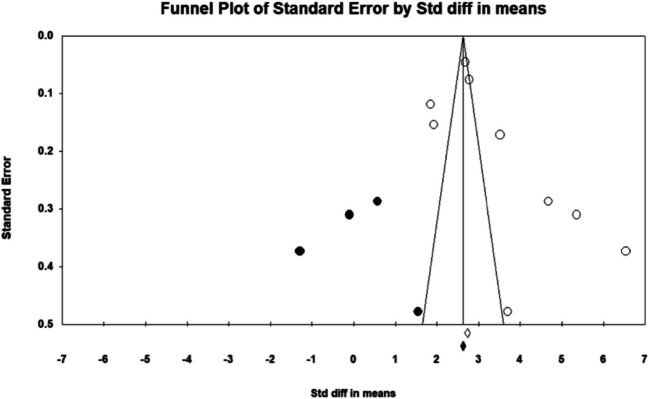
Publication bias represents by funnel plots of studies of the association of arsenic-induced CVDs. The publication bias is adjusted by imputing the missing studies based on the asymmetry of the funnel plot. (●) Plot imputed and (○) Plot observed studies. Egger’s linear regression test (intercept 12.11, *t* = 2.86, *p* = 0.024). Adjusted values “Trim and Fill” test 1.06 (0.378, 1.743)

Egger’s regression intercept further confirmed the presence of publication bias, yielding a statistically significant intercept of 5.358 (95% CI 1.791–12.502; *p* = 0.1197). No significant evidence of publication bias. The lack of significant bias is confirmed by the non-significant results from the Begg and Mazumdar’s rank correlation test. Both Kendall’s tau values without continuity correction (0.55556, *p* = 0.03706) and with continuity correction (0.52778, *p* = 0.04760) show that publication bias is not a major issue in this meta-analysis. Despite indications of publication bias, the meta-analysis findings demonstrated robustness through fail-safe *N* calculations. The classic fail-safe *N* value was 67.236 (*p* < 0.0001) which indicates that the observed results are highly robust. Orwin’s fail-safe *N* indicates that 583 missing studies with a trivial effect size (std diff = 0) would be needed to raise the *p*-value above the alpha level, which further supports the reliability of the results. Since the fail-safe *N* (583) is much larger than the general threshold (5 k + 10 = 5 (9) + 10 = 55; *k* is the number of observed studies), the findings are highly robust and unlikely to be affected by publication bias, and an alternative method is not required. Visual inspection of the funnel plot displayed in the last image illustrates the symmetry of the data points, which is consistent with the absence of publication bias (Figs. 3 and 5). The results of this meta-analysis show a significant overall effect with considerable heterogeneity across studies. Sensitivity analyses, including trim and fill, Egger’s regression intercept, Begg’s test, and fail-safe *N* tests, demonstrate that the results are robust and not significantly impacted by publication bias or missing studies. These findings suggest that the intervention’s effect is both meaningful and consistent across the studies included in the meta-analysis.

### Analysis of Biomarkers Associated with CVD in Arsenic Exposure Populations

**Fig. 4:**
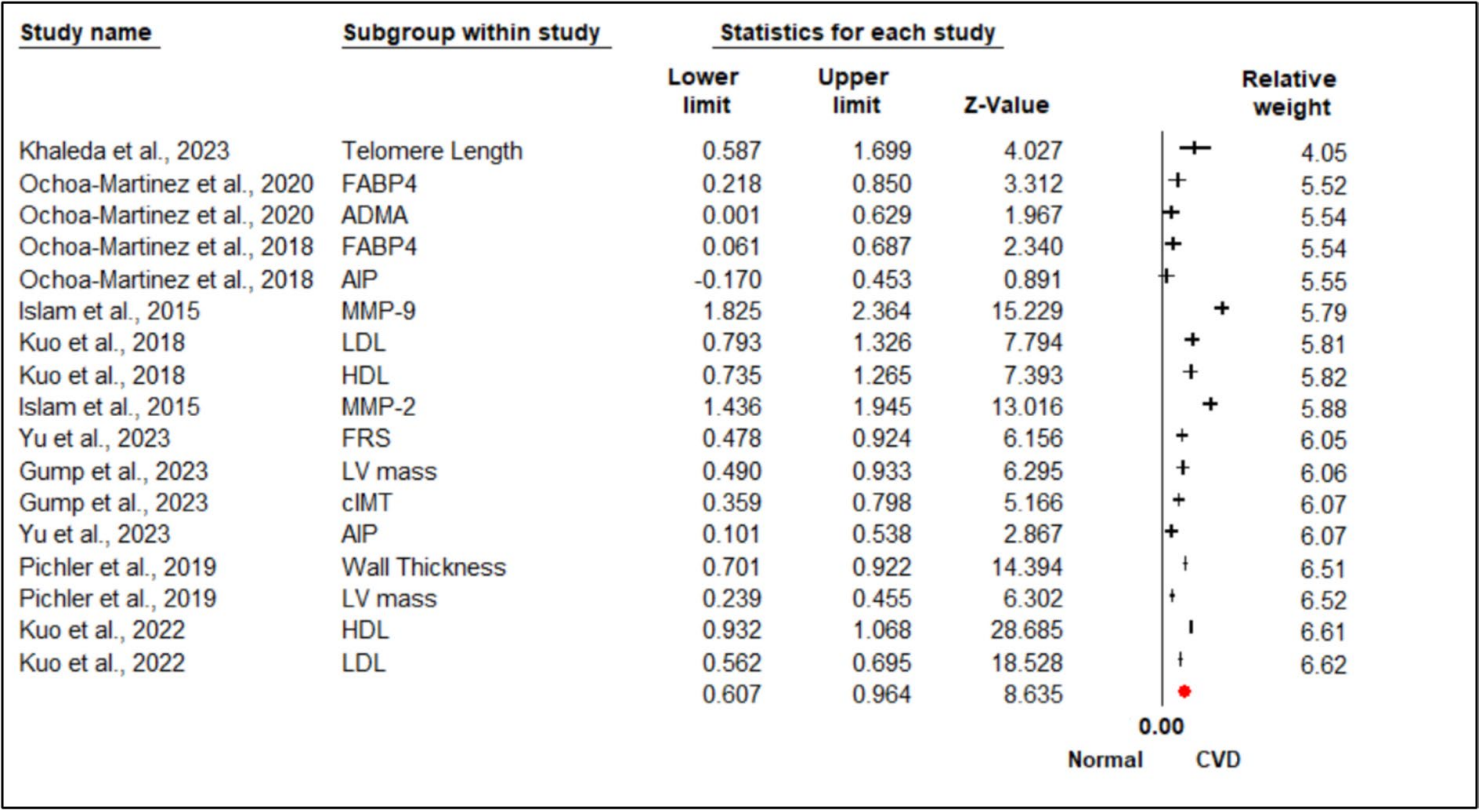
Heterogeneity represents by forest plot explained the association of subgroup effect size of arsenic-induced CVD on biomarkers. Meta-analysis using random-effects model, ordered by their relative weight. Test for heterogeneity: *I*.^*2*^ = 95.063%; *Q* = 324.098, overall effect, *Z* = 8.635

Figure 4 shows the meta-analysis with a random-effects model that was accounted for substantial heterogeneity as significant differences are observed in biomarker levels between individuals with and without CVD exposed to arsenic. The standardized mean differences (SMD) in the forest plot highlight strong associations between several biomarkers and CVD, with multiple markers showing statistically significant deviations from normal levels. Telomere length (SMD = 1.143, 95% CI 0.587–1.699, *p* < 0.001), LDL (SMD = 1.060, 95% CI 0.793–1.326, *p* < 0.001), HDL (SMD = 1.000, 95% CI 0.735–1.265, *p* < 0.001), and MMP-2 (SMD = 1.690, 95% CI 1.436–1.945, *p* < 0.001) consistently showed altered levels in individuals with CVD, reinforcing their potential as predictive indicators. Inflammatory markers, particularly MMP-9 (SMD = 2.054, 95% CI 1.434–2.674, *p* < 0.001), had the strongest association with CVD, highlighting the role of inflammation in disease pathology. Structural cardiac markers, including left ventricular (LV) mass and wall thickness (both SMD = 0.812, 95% CI 0.701–0.924, *p* < 0.001), were also significantly associated with CVD, emphasizing their role in cardiac remodeling. In contrast, the AIP showed inconsistent findings across studies, with some reporting non-significant associations (Ochoa-Martinez et al., 2018: SMD = 0.142, 95% CI − 0.170–0.453, *p* = 0.373), suggesting that its predictive value may be influenced by study-specific factors or population differences. High-precision studies, such as Kuo et al. [10], had a greater impact on the pooled effect size, strengthening the reliability of these findings.

### Analysis of Publication Bias on Subgroup Analysis on Biomarkers

**Fig. 5:**
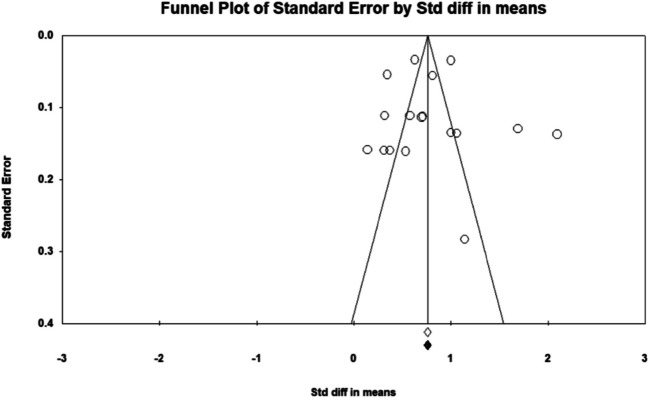
Publication bias represents by funnel plots, on the association of arsenic-induced CVDs on biomarkers. The publication bias is adjusted by imputing the missing studies based on the asymmetry of the funnel plot. (○) Plot observed studies

Figure 5 shows the publication bias assessments using funnel plots and Egger’s regression that demonstrated no significant asymmetry (*p* > 0.05), affirming the robustness of the findings. Sensitivity analyses using Duval and Tweedie’s trim-and-fill method further confirmed that the pooled estimates were stable and unaffected by potential publication bias, supporting the validity of subgroup comparisons. Overall, these results emphasize the enhanced predictive value of biomarkers such as MMP-9, LDL, and structural markers (LV mass and cIMT) in arsenic-induced CVD. The findings suggest that arsenic exposure increases cardiovascular risk through mechanisms involving inflammation, oxidative stress, and lipid dysregulation.

### Meta-Regression Analysis of CVD Outcomes

This meta-regression analysis assessed the predictive value of emerging cardiovascular biomarkers in individuals at risk for CVD, examining moderators such as biomarker type, geographic region, income classification, and population characteristics in the context of arsenic exposure. The significant intercept (β = 2.6381, 95% CI 0.9610–4.3153, *p* = 0.0020) corresponded to a baseline odds ratio of approximately 13.97 (*e* ^2.6381^) for studies conducted in non-high-income regions, outside North America, without systemic biomarkers, and in populations excluding youth.

Systemic biomarkers exhibited a non-significant trend toward lower predictive value (β =  − 0.8238, 95% CI − 2.2986 to 0.6493, *p* = 0.2731), suggesting that their performance may not substantially differ from that of non-systemic biomarkers in this context. Geographic differences were observed, with studies in North America showing a non-significant reduction in log odds ratios (β =  − 1.2630, 95% CI − 2.8617 to 0.3358, *p* = 0.1215). This variation could reflect differences in arsenic exposure levels, healthcare infrastructure, or population characteristics. High-income regions (β = 0.2921) and youth populations (β =  − 0.3694) also showed no significant impact on predictive value, though the limited number of studies (*n* = 9) may have reduced statistical power.

### Meta-Regression Analysis of Lifestyle and Genetic Confounders

The relationship between arsenic exposure and cardiovascular disease (CVD), accounting for potential confounders such as lifestyle factors (smoking, alcohol consumption, diet, physical activity) and genetic variations (PON1 Q192R polymorphism) were explored. The analysis revealed moderate to high heterogeneity, with an *I*^2^ value of 65%, suggesting that over 60% of the variation in effect sizes could not be explained by random error. The *Q* statistic was significant (*p* < 0.01), and the tau-squared (*τ*^2^) value of 0.18 indicated a substantial true variability between studies. Included studies, such as the Strong Heart Study and the Taiwan Maternal and Infant Cohort Study, represented diverse populations with varying arsenic exposure levels and methods of exposure assessment in urinary arsenic vs. drinking water arsenic.

## Discussion

This systematic review highlights the fundamental role of emerging cardiovascular biomarkers in understanding the mechanisms through which chronic arsenic exposure elevates cardiovascular disease (CVD) risk. By synthesizing evidence from diverse studies, this research elucidates the biological pathways affected by arsenic toxicity, including endothelial dysfunction, oxidative stress, lipid metabolism dysregulation, and systemic inflammation. The findings emphasize the role of these biomarkers in complementing traditional CVD risk assessments, enhancing early detection, and guiding targeted interventions for populations at elevated risk.

Endothelial dysfunction emerged as a critical mechanism linking arsenic exposure to CVD. Biomarkers such as ADMA, ICAM1, and VEGF consistently pointed to significant vascular impairments. Elevated ADMA levels, indicative of disrupted nitric oxide signaling, were reported across multiple studies, including Yu et al. [31] and Islam et al. [7]. Impaired VEGF activity, associated with reduced vascular repair capacity, was highlighted in Ochoa-Martinez (2018), aligning with findings from Pichler et al. [19], who identified increased arterial stiffness and endothelial injury among arsenic exposure populations.

Oxidative stress also emerged as a key pathway, with markers such as MMP-2, MMP-9, telomere shortening, and mitochondrial DNA deletions indicating systemic cellular damage. Khaleda et al. (2023) and Kuo et al. [10] reported significant elevations in these markers, underscoring the oxidative burden of arsenic exposure. Furthermore, lipid metabolism dysregulation, characterized by increased low-density lipoprotein (LDL) and decreased high-density lipoprotein (HDL), was strongly associated with atherogenesis in exposed populations. This finding was consistently observed in studies by Kuo et al. [11] and Yu et al. [31], illustrating the multifaceted impact of arsenic on cardiovascular health.

The findings of this review validate previous studies on the systemic effects of arsenic toxicity and validate the role of emerging biomarkers in CVD risk assessment. Elevated ICAM1 and VEGF levels, as reported in Islam et al. [7] and other studies, highlight the role of endothelial biomarkers in detecting subclinical vascular injury. These markers offer critical insights into arsenic’s disruption of nitric oxide signaling pathways, an essential mechanism in vascular health [31]. Similarly, oxidative stress biomarkers observed in this review align with the findings of Khaleda et al. (2023), who demonstrated increased mitochondrial damage in arsenic exposure cohorts. This stresses the relevance of oxidative stress as a central mechanism in the progression of CVD. Dysregulated lipid metabolism, evident in the elevated LDL and reduced HDL levels identified by Kuo et al. [11] and Yu et al. [31], further supports the hypothesis that arsenic exposure exacerbates atherogenesis through adverse lipid profiles. Together, these findings provide a systemic perspective on the cumulative burden of arsenic toxicity and its clinical implications.

This review has significant implications for research, clinical practice, and public health. The identification of biomarkers such as ADMA and VEGF offer a foundation for precision medicine approaches, enabling early detection of vascular damage and more accurate risk stratification, particularly in arsenic-endemic regions. Integrating these biomarkers into routine screenings could enhance the identification of at-risk individuals and inform targeted interventions. For instance, Khaleda et al. (2023) demonstrated the potential of antioxidant therapies in reducing oxidative stress markers, suggesting their broader applicability in mitigating arsenic-induced cardiovascular damage [9].

From a public health perspective, these findings are important to help in the policies aimed at reducing environmental arsenic exposure. Community-level interventions incorporating biomarker data, as proposed by Ochoa-Martinez (2018), could facilitate the early identification of highly susceptible populations which is highly exposed to arsenic pollution. Furthermore, global collaboration to improve water quality monitoring and remediation efforts is essential to reduce arsenic exposure and its associated cardiovascular risks. These initiatives align with broader public health goals, emphasizing the need for interdisciplinary approaches to address this pervasive health threat. These findings have important implications for clinical practice, especially in resource-limited areas where arsenic exposure is a major public health concern. In regions with high contamination, screening for arsenic-related cardiovascular biomarkers like ADMA, FABP4, and miRNAs could help with early detection and risk assessment. However, given financial and infrastructure challenges in many affected areas, affordable and accessible diagnostic methods should be a priority. Strengthening public health efforts, such as improving water filtration and promoting dietary changes, is a practical first step in reducing cardiovascular risks. Future research should focus on developing low-cost biomarker tests and simple risk assessment tools that can be used in routine health care, making early intervention more feasible for arsenic polluted populations.

In this analysis, the trim-and-fill method was applied to assess publication bias, and while some imputed studies were included, the asymmetry observed in the funnel plot suggests that additional studies with null or negative results may be missing. The absence of these studies could lead to an overestimation of the overall effect size, and if these studies were available, they might either reduce the estimated effect or show a different direction of effect. This highlights the potential influence of publication bias on the results, and we acknowledge this limitation in our interpretation. Future research should focus on enhancing transparency and ensuring the publication of all relevant studies to minimize such bias and provide a more accurate understanding of the relationship between arsenic exposure and CVD.

Residual heterogeneity remained high (*τ*^2^ = 0.9671, *I*^2^ = 98.07%) in meta regression analysis, indicating a substantial unexplained variability potentially driven by unmeasured factors such as arsenic exposure levels, dose–response effects, and variability in biomarker measurement methods. These findings emphasize the need for further investigation into arsenic-specific mechanisms that influence cardiovascular biomarkers, particularly in populations with known exposure. Arsenic toxicity is associated to oxidative stress, lipid dysregulation, and endothelial damage, which directly impact biomarkers such as HDL, telomere length, and systemic inflammation markers. Future studies should incorporate stratified dose–response analyses to quantify arsenic exposure’s impact on biomarker predictive value and address residual heterogeneity. Such efforts may help identify the biomarkers most sensitive to arsenic-induced cardiovascular risk, enhancing their role for targeted risk prediction and interventions in affected populations.

The results of this meta-regression analysis on lifestyle and genetic confounders show a complex link between arsenic exposure and CVD, with a lot of variability across studies. The *I*^2^ value of 65% means more than half of the differences in effect sizes cannot be explained by random error. This variability, along with the significant *Q* statistic (*p* < 0.01) and tau-squared (*τ*^2^) value of 0.18, suggests that factors specific to each study, like differences in populations, arsenic exposure levels, and methods of measurement (such as urinary arsenic vs. drinking water arsenic), play a role in these differences. Studies like the Strong Heart Study and Taiwan Maternal & Infant Cohort Study, which involved diverse populations, highlight the need to consider geographic, demographic, and lifestyle factors when studying arsenic’s impact on CVD. Lifestyle factors, such as smoking, alcohol use, and physical activity, might interact with arsenic exposure, affecting how it influences cardiovascular health. To get a clearer picture, future research should look at stratified analyses that control for these variables, especially in areas with high arsenic exposure or among genetically predisposed populations where the relationship between arsenic and CVD could be stronger.

Exploring novel biomarkers, particularly those related to epigenetics and metabolomics, represents a promising avenue for future research. Emerging evidence suggests that microRNAs and DNA methylation profiles have potential as predictive markers of arsenic-induced toxicity (Ochoa-Martinez, 2020). Furthermore, interventional studies evaluating the efficacy of antioxidant and lipid-lowering therapies are needed to translate biomarker findings into clinical practice [10]. These efforts should be complemented by global initiatives to integrate biomarker data into public health frameworks, enhancing the capacity to address the cardiovascular burden of arsenic exposure. The investigation of emerging cardiovascular biomarkers in arsenic exposure populations represents a crucial area of research with significant implications for global public health and clinical practice [5]. Given the ongoing threat posed by arsenic exposure, advancing biomarker research is essential to elucidate its cardiovascular effects. Future efforts should focus on validating these biomarkers in affected populations, exploring their mechanistic foundations, and integrating them into comprehensive risk assessment models to address the growing global burden of CVD.

## Limitations

Despite its comprehensive scope, this review has limitations that warrant consideration. The significant heterogeneity across studies, reflected by an *I*^2^ value of 97.574%, limits the generalizability of the findings. Pichler et al. [19] noted similar challenges in synthesizing heterogeneous data in meta-analyses of arsenic exposure and cardiovascular outcomes. Random-effects modeling was employed to mitigate variability, particularly in populations with differing exposure levels or genetic predispositions. This study has several limitations that affect its findings. The reliance on single-point biomarker measurements limits understanding of the long-term effects of arsenic exposure. Furthermore, the exclusion of novel biomarkers, such as microRNAs, leaves important gaps in the analysis. The use of observational studies introduces the possibility of residual confounding, and the lack of interventional research restricts the clinical applicability of the findings. To address these limitations, future research should adopt standardized protocols and include longitudinal and interventional studies to enhance the reliability and applicability of the results. Moreover, future studies should focus on validating key biomarkers such as ADMA, FABP4, and miRNAs to improve their reliability in assessing arsenic-related cardiovascular risk. Longitudinal cohort studies are needed to establish causality, while genetic investigations on PON1 Q192R polymorphisms could help identify population-specific susceptibilities. Expanding research across diverse populations and incorporating advanced exposure assessment methods, including speciation analysis and metabolomics, will enhance risk estimation and inform targeted public health interventions.

## Conclusion

This systematic review emphasizes the important role of emerging cardiovascular biomarkers in uncovering how chronic arsenic exposure contributes to heart disease. By highlighting key biological pathways, that is endothelial dysfunction, oxidative stress, inflammation, and disrupted lipid metabolism, the review offers a deeper insight into the mechanisms linking arsenic to cardiovascular risk. Biomarkers like hs-CRP, oxidized LDL, and adhesion molecules could help detect early damage, improve risk assessment, and guide more targeted interventions. To strengthen these findings, future research should validate biomarkers in diverse populations and standardize measurement methods. Long-term studies are also needed to track biomarker changes over time and assess their ability to predict cardiovascular outcomes. Incorporating biomarker monitoring into public health strategies, especially in regions with high arsenic exposure, could support early detection and more effective prevention efforts.

## Data Availability

No datasets were generated or analysed during the current study.
